# Brief alcohol training for psychiatric staff

**DOI:** 10.1186/1940-0640-7-S1-A63

**Published:** 2012-10-09

**Authors:** Christina Nehlin, Anders Fredriksson, Leif Grönbladh, Lennart Jansson

**Affiliations:** 1 Department of Neuroscience/Psychiatry, Uppsala University, Uppsala, Sweden

## 

Staff attitudes are crucial to the successful implementation of systematic alcohol screening and brief intervention (SBI). The forms and extent of training needed to improve attitudes among psychiatric staff with regard to problem drinking is unclear. In this study, we assumed psychiatric staff to be familiar with alcohol as a problem area and therefore tested briefest possible training effort. This study was conducted as part of a national Swedish project aimed at promoting the use of SBI within psychiatric care. The aim of the study was to study psychiatric staff’s knowledge and attitudes to problem drinking. A further aim was to investigate whether three hours of training in a single session would be sufficient to improve knowledge and therapeutic attitudes toward problem drinking. A tailored training model for nonphysician psychiatric staff was carried out at a medium-sized university clinic. Participants included nurses and psychiatric aides (medical) and psychologists and social workers (nonmedical). The training consisted of a two-hour workshop and a one-hour follow-up session. Staff knowledge and attitudes to problem drinking patients were measured at baseline and at follow-up by case-vignette assessment and by administration of the Short Alcohol and Alcohol Problems Perception Questionnaire (SAAPPQ). In total, 115 persons completed the questionnaire (follow-up rate = 83%). Distribution of medical and nonmedical staff was 50/50. After training, nonmedical staff estimated vignette case severity higher than before training. Both groups had a higher estimate of their capacity to help a patient with alcohol problems. Self-rated role adequacy was higher in both groups. Medical staff also scored higher on work satisfaction. We conclude that three hours of tailored training improved psychiatric-staff knowledge and therapeutic attitudes toward problem drinking. Nonmedical staff in particular benefitted from the training.

